# A Comparison of Tumor Biology in Primary Ductal Carcinoma *In Situ* Recurring as Invasive Carcinoma versus a New *In Situ*

**DOI:** 10.1155/2013/582134

**Published:** 2013-12-29

**Authors:** Wenjing Zhou, Christine Johansson, Karin Jirström, Anita Ringberg, Carl Blomqvist, Rose-Marie Amini, Marie-Louise Fjallskog, Fredrik Wärnberg

**Affiliations:** ^1^Department of Surgery, Uppsala University, Uppsala, Sweden; ^2^Department of Immunology, Genetics and Pathology, Uppsala University, Uppsala, Sweden; ^3^Department of Clinical Sciences, Pathology, Lund University, Lund, Sweden; ^4^Department of Plastic and Reconstructive Surgery, Malmö Hospital, Lund University, Lund, Sweden; ^5^Department of Oncology, Helsinki University Central Hospital, Helsinki, Finland; ^6^Department of Radiology, Oncology and Radiation Science, Uppsala University, Uppsala, Sweden

## Abstract

*Introduction*. About half of all new ipsilateral events after a primary ductal carcinoma *in situ* (DCIS) are invasive carcinoma. We studied tumor markers in the primary DCIS in relation to type of event (invasive versus *in situ*). *Methods*. Two hundred and sixty-six women with a primary DCIS from two source populations, all with a known ipsilateral event, were included. All new events were regarded as recurrences. Patient and primary tumor characteristics (estrogen receptor (ER), progesterone receptor (PR), HER2, EGFR, and Ki67) were evaluated. Logistic regression was used to calculate odd ratios and 95% confidence intervals in univariate and multivariate analyses. *Results*. One hundred and thirty-six of the recurrences were invasive carcinoma and 130 were *in situ*. The recurrence was more often invasive if the primary DCIS was ER+ (OR 2.5, 95% CI 1.2–5.1). Primary DCIS being HER2+ (OR 0.5, 95% CI 0.3–0.9), EGFR+ (OR 0.4, 95% CI 0.2–0.9), and ER95−/HER2+ (OR 0.2, 95% CI 0.1–0.6) had a lower risk of a recurrence being invasive. *Conclusions*. In this study, comparing type of recurrence after a DCIS showed that the ER−/HER2+ tumors were related to a recurrence being a new DCIS. And surprisingly, tumors being ER+, HER2−, and EGFR− were related to a recurrence being invasive cancer.

## 1. Introduction

Ductal carcinoma *in situ* (DCIS) of the breast is a clinically and molecularly heterogeneous disease with different malignant potentials [1, 2]. The risk of local recurrence after breast-conserving surgery only (BCS) is rather high and even higher than after surgery for invasive breast cancer [3, 4]. In DCIS, adding radiotherapy after BCS lowered the relative risk with approximately 50%, from 28.1% to 12.9% after ten years in a meta-analyses including four randomized studies [5, 6]. About half of the women with a local recurrence develop a new DCIS and the other half an invasive carcinoma [7–10]. Although women with a primary DCIS have a very good prognosis [6], those with a subsequent invasive carcinoma have an increased risk of dying from breast cancer. In a recently published study the 15-year breast cancer specific survival was just over 60% among those with an invasive recurrence after a primary DCIS [11, 12].

One of the major goals of the treatment of DCIS is to prevent invasive disease. There are surgical and biological risk factors for local recurrence, for example, young age, mode of detection (clinically detected as compared to screening detected), margins, and grade [13, 14]. Also, a recently published study using the Oncotype DX DCIS score showed that genomic-based data could predict local relapse risk independently from classical factors [15]. The expression of estrogen receptors (ER), progesterone receptors (PR), and human epidermal growth factor receptor-2 (HER2) has been shown to predict local recurrence [16–19]. However, little is known about factors associated with the risk of developing invasive breast cancer.

The aim of this study was to look for patient and primary tumor characteristics that might be associated with progression from *in situ* to invasive breast cancer. If we can predict who has a low risk for an invasive recurrence, this might help us to individualize type of surgery and adjuvant treatment, but also it is interesting for our knowledge in cancer progression in general. We studied a large number of women with a known local recurrence after a primary DCIS and compared the expression of specific biomarkers and other patient characteristics in those with an *in situ* and those with an invasive recurrence, respectively. This paper does not look at risk of recurrence, as we did not have the background information for women without a new ipsilateral event in all women.

## 2. Methods

### 2.1. Patients

Patients were recruited from two different source populations. One was a population-based cohort comprising all 458 women diagnosed with a primary DCIS between 1986 and 2004 in the Uppland and Västmanland regions of Sweden. All women with a recurrence (*n* = 100) up to December 31, 2008, were included. The other source was the SweDCIS trial which was a randomized multicentre trial consisting of 1,046 women diagnosed between 1987 and 1999 with a primary DCIS, administered through the Regional Oncological Centres in all six Swedish Health Care Regions. Patients included in the SweDCIS had a diagnosis of primary DCIS, occupying less than one-quadrant of the breast, and underwent surgery with breast conservation. After surgery, the women were randomized to receive postoperative radiotherapy of the breast or not. We included all women from the study with a registered local recurrence (*n* = 166) up to December 31, 2005. All new ipsilateral breast cancer events were considered a local recurrence and the earliest event considered a recurrence occurred after seven months.

### 2.2. Tissue Microarray (TMA) Construction

Prior to the TMA construction, H&E sections from all paraffin blocks from the primary DCIS cases were histopathologically reevaluated and graded by one pathologist (KJ) and appropriate tumor areas selected. Two cores with a diameter of 1.0 mm were mounted into the recipient TMA block using a manual arraying device (MTA-1, Beecher Inc., WI, USA). The concordance of immunohistochemical (IHC) staining between biopsies from the same lesion in DCIS and between original whole section slides and TMA-slides has previously been and reported. [20, 21]. The concordance was 80.4% for HER2, 84.2% for ER, and 81.5% for PR.

### 2.3. IHC and Silver-Enhanced *In Situ* Hybridization (SISH)

We performed IHC for ER, PR, HER2, epidermal growth factor receptor (EGFR), cytokeratin 5/6 (CK5/6), and Ki67 on 4 *μ*m paraffin sections cut from the TMAs. Immunostains for each marker were performed on a Dako Autostainer (Dako Corporation). IHC was conducted according to established protocols. Appropriate positive and negative controls were included in all staining runs. The antibodies used were c-erbB-2 poly rabbit, A0485, DAKO, USA, ER NCL-6F11, Novocastra, UK, and PgR NCL-1A6, Novocastra, UK, Ki-67 MIB-1, Immunotech, KEBO, CK5/6, D5/16B4, Zymed, USA, and EGFR 31G7, Zymed, USA.

HER2 SISH was also performed on TMA slides using an automated instrument, Ventana Benchmark (Ventana Medical Systems, Tucson, AZ, USA), as per the manufacturer's protocols for the INFORM HER2 DNA probe and chromosome 17 probes. Testing for the HER2 gene and chromosome 17 was performed on sequential sections. Both probes were labeled with dinitrophenol. Denaturation occurred on the instrument with enzyme digestion in protease 3 for 8 minutes. The detection system used a multimer labeled with goat anti-rabbit antibody horseradish peroxidase as the linking step. Visualization occurred with the sequential addition of silver acetate as the source of ionic silver, hydroquinone, and hydrogen peroxide to give a black metallic silver precipitate at the probe site. Counterstaining was performed with hematoxylin II on the instrument. The time taken for the complete run was 6.5 hours. Both HER2 and chromosome 17 detection were performed on the same slide run. Gene amplification was assessed using the American Society of Clinical Oncology/College of American Pathologists guideline and Australian HER2 Advisory Board criteria for single HER2 probe testing (diploid 1 to 2.5 copies/nucleus; polysomy >2.5 to 4 copies/nucleus; equivocal >4 to 6 copies/nucleus; low-level amplification >6 to 10 copies/nucleus; and high-level amplification >10 copies/nucleus) and for dual HER2/CHR17 probe testing (nonamplified ratio <1.8; equivocal ratio 1.8 to 2.2; gene amplification >2.2). The status of HER2 protein expression was firstly assessed using SISH and secondly, for those cases on which SISH failed, the evaluation was based on IHC.

### 2.4. Scoring and Classification

Data on tumor size and multifocality was obtained from the original histopathological reports. Stained TMA slides were scanned (ScanScope XT, Aperio, USA) for evaluation of expression of ER, PR, HER2, EGFR, CK 5/6, and Ki67 by ImageScope (Aperio, USA). Tumors that showed nuclear staining more than 10% were considered ER or PR positive, as this was and still is the routinely used cutoff in Sweden. Using the HerceptTest classification system, tumors were considered HER2 positive if the score was 3+. Any degree of cytoplasmic staining for CK 5/6 and any degree of distinct membranous staining for EGFR were counted as positive, even if focal. CK 5/6 and EGFR were used to define an ER−/HER2− tumor as basal like according to the classification system by Livasy et al. [22]. Proliferation was considered high if immunostaining for Ki67 was seen in more than 10% of tumor nuclei. These latter IHC criteria are similar to those previously used for scoring these markers in invasive breast cancer [22–27]. If only one core included enough tumor tissue, this was used for classification but at least 200 cells had to be counted. Each marker was scored by one person blinded for outcome (WZ or CJ). HER2 SISH was scored by WZ with RMA as a reference. The recurrences were defined as invasive or *in situ* based on the original histopathological report. We did not have data on ER, PR, or HER2 for the new events.

### 2.5. Statistical Analyses

Among those primary DCIS with a recurrence, the associations between baseline characteristics and type of recurrence (invasiveor *in situ*) were analyzed using logistic regression models. Odds ratio (OR) and 95% confidence interval (95% CI) were used to estimate the relative risks. In the multivariate models, we adjusted for age group, free margins, and type of surgery. Data analyses were conducted using the SAS System  9.2 (SAS Institute, NC, USA).

The guidelines for tumor marker prognostic studies (reporting of tumor MARKer studies (REMARK)) including relevant items about test evaluation were followed [28]. This study was approved by the Ethics Committee at Uppsala University Hospital (Dnr 2005: 118) and Umeå University (Dnr 05-065 M).

## 3. Results

Of the 1,504 (458 + 1,046) women with a primary DCIS, 136 developed an invasive recurrence and 130 developed an *in situ* recurrence. Baseline clinical and histopathological characteristics for the two groups (DCIS with an invasive recurrence and DCIS with an* in situ *recurrence) are presented in Tables 1 and 2. Age at diagnosis was comparable between the groups. Time to an *in situ* recurrence was on average 44 months and to an invasive recurrence was 67 months. No women in this study received pre- or postoperative chemotherapy or endocrine treatment. The analyses were done in the two different source populations separately and then pooled together and the results did not differ substantially except for nuclear grade (NG) (Table 3). All analyses were also performed restricted to patients receiving postoperative radiotherapy, to patients not receiving radiotherapy, and to patients undergoing BCS only, respectively. The results did not differ substantially from the analyses including all patients and data are not presented.

The ER+ tumors were of NG1 in 11.7%, NG2 in 40.1%, and NG3 in 48.2% of the cases, compared to 2.7%, 18.9%, and 78.4%, respectively, for the ER− tumors (*P* = 0.004). Among the HER2+ tumors 1.5% were of NG1, 19.7% of NG2, and 78.8% of NG3 compared to 13.6%, 50.5%, and 35.9%, respectively, for the HER2− tumors (*P* < 0.0001).

### 3.1. Results of Factors Associated with Subsequent Invasive Cancer versus DCIS

Clinically detected DCIS lesions with a known recurrence were associated with a higher risk of the recurrence being invasive (OR 1.80, 95% CI 1.02–3.19) compared to those DCIS detected by mammography screening (Table 3). Large size in the primary DCIS (>15 mm or multifocality) was associated with a lower risk of a recurrence being invasive (OR 0.54, 95% CI 0.32–0.92). Type of surgery, involvement of margins, and NG were not statistically significantly associated with type of recurrence.

ER+ primary DCIS with a known recurrence had a higher risk of the recurrence being invasive (OR 2.52, 95% CI 1.24–5.10). HER2+ and EGFR+ primary DCIS tumors with a known recurrence were associated with a halved risk of the recurrence being invasive OR 0.48, 95% CI 0.26–0.90 and OR 0.44, CI 0.22–0.88, respectively. The ER−/HER2+ tumors, with the ER+/HER2− tumors as a reference, were associated with a lower risk of the recurrence being invasive (OR 0.24, 95% CI 0.09–0.62), while the other subgroups were not statistically significantly associated with type of recurrence. Other molecular factors including PR, CK 5/6, and Ki67 were not statistically significantly associated with type of recurrence (Table 3).

## 4. Discussion

In this study, the purpose was to study patient and tumor characteristics in women with a primary DCIS followed by a recurrence and to compare primary tumors recurring as invasive carcinoma with those recurring as DCIS. Surprisingly, we found that ER+, HER2−, and EGFR− tumors were strongly associated with a subsequent recurrence being invasive.

Usually, studies are designed to evaluate the risk of recurrence in relation to different tumor markers, patient characteristics, or type of treatment. In this study setting we chose to only include DCIS with a known recurrence. Hypothetically, we had two comparable groups of DCIS that recurred either as invasive carcinoma or DCIS. Differences found regarding tumor biology related to type of recurrence can possibly reflect a true potential to progress from *in situ* to invasive carcinoma. Of course, we cannot rule out the possibility of some of the tumors being new cancers instead of true recurrences.

We collected a large number of primary DCIS with a documented recurrence and constructed TMAs. TMA construction from DCIS is somewhat challenging due to the often relatively small and scattered lesions, and there will always be a selection bias regarding tumor size. Additionally, the loss of representative tumor material for each subsequent section cut tends to increase. This resulted in missing IHC data in about 30% for ER that was stained for in the first section and in nearly 50% of the cases for EGFR and Ki67 that were stained for in the last sections.

Nuclear grade and age at diagnosis were not associated to type of recurrence in our study. Clinically detected DCIS showed a higher risk for a recurrence being invasive. This finding is consistent with the observations that clinically detected invasive breast cancers tend to be more aggressive than lesions detected by mammography screening [17, 29]. Large tumor size was associated to recurrences being of the *in situ *type after adjusting for age, margins, and type of surgery in the multivariate analysis. This might be due to a higher risk of residual *in situ* after surgery in larger lesions.

We found that a certain combination of molecular markers ER−/HER2+ was statistically significantly associated with a high risk for a recurrence being *in situ*. This finding is consistent with the results from one previous, nested case-control study, showing that ER−/HER2+ DCIS was associated with an increased risk of recurrent DCIS, but not with the risk of invasive recurrence [17].

We found that women with HER2+ tumors had a higher risk of a recurrence being of the *in situ* type. Recently, Rakovitch et al. [30] observed that women with a HER2+/Ki67+ DCIS had a higher risk of developing *in situ* local recurrence after breast-conserving surgery which is consistent with our results regarding HER2 status. HER2 is one of the most extensively studied biological prognostic factors in invasive breast cancer. However, its importance in DCIS has yet to be elucidated [31]. There are three published studies where no significant associations were found between a variety of biologic markers, including HER2, and the risk of recurrence after a DCIS [16, 31, 32]. In contrast to those studies, we used a stringent cutoff for HER2 positivity. We used SISH when possible (*n* = 162) and secondly IHC (3+) to determine HER2 positivity. Hence, it is still possible that high-level HER2 overexpression due to gene amplification does predict recurrence after a primary DCIS but the tumor biology explaining why recurrences after a HER2+ DCIS more often are of the *in situ* type remains to be explored.

EGFR, like HER2, is a potent stimulating factor of cell-growth-activating pathways and thus stimulates tumor growth when activated [33]. EGFR has been used as a surrogate marker for basal like invasive breast cancer and for DCIS [22, 26]. EGFR overexpression has been associated with a poor outcome in invasive breast cancer but very little is reported on DCIS [34–36]. In our study, EGFR positivity was associated with a higher risk for a recurrence being of the *in situ* type, similar to the recurrences after HER2+ DCIS.

In conclusion, given that a woman experienced a recurrence after a primary DCIS, tumor markers related to a recurrence being invasive compared to a new *in situ* were ER+, HER2−, and EGFR−. This marker profile might signal a potential for a DCIS to progress from *in situ* to invasive carcinoma.

## Figures and Tables

**Table 1:** Baseline clinical and histopathological characteristics among women with a primary DC44IS who later developed either an invasive cancer or an *in situ* recurrence. Women with a known recurrence were recruited from two source populations: a population based cohort (U/V cohort, *n* = 458) and a randomized study (SweDCIS, *n* = 1,046).

Baseline characteristics at diagnosis of primary DCIS	All DCIS with a recurrence (*n* = 266)					
	U/V cohort (*n* = 100) Type of recurrence		SweDCIS (*n* = 166) Type of recurrence		All DCIS with a recurrence (*n* = 266) Type of recurrence	
	Invasive (*n* = 55)	*In situ* (*n* = 45)	Invasive (*n* = 81)	*In situ* (*n* = 85)	Invasive (*n* = 136)	*In situ * (*n* = 130)
	Number	Number	Number	Number	Number (%)	Number (%)
Time to recurrence, months (mean ± SD)	65 ± 44	54 ± 49	67 ± 43	37 ± 27	67 ± 44	44 ± 37
Age at diagnose (*n* = 266)						
≤45	9	7	16	14	25 (18.4)	21 (16.2)
46–60	22	22	36	39	58 (42.7)	61 (46.9)
>60	24	16	29	32	53 (38.9)	48 (36.9)
Mode of detection (*n* = 265)						
Screening	37	36	53	65	90 (66.7)	101 (77.7)
Clinically	18	9	27	20	45 (33.3)	29 (22.3)
Tumor size (*n* = 234)						
≤15 mm	34	16	40	41	74 (63.2)	57 (48.7)
>15 mm or multifocal	17	24	26	36	43 (36.8)	60 (51.3)
Type of surgery (*n* = 266)						
Breast conserving surgery	50	41	81	85	131 (96.3)	126 (96.9)
Mastectomy	5	4	—	—	5 (3.7)	4 (3.1)
Postoperative radiotherapy (*n* = 266)						
Yes	14	12	27	20	41 (30.2)	32 (24.6)
No	41	33	54	65	95 (69.8)	98 (75.4)
Free margins (*n* = 255)						
Yes	47	34	61	65	108 (80.0)	99 (76.2)
No or doubtful	8	11	19	20	27 (20.0)	31 (23.9)
Nuclear grade (*n* = 241)						
I	5	6	6	1	11 (9.2)	7 (5.8)
II	19	17	26	21	45 (37.5)	38 (31.4)
III	26	21	38	55	64 (53.3)	76 (62.8)

**Table 2:** Molecular characteristics among women with a primary DCIS who later developed either an invasive cancer or an *in situ* recurrence. Women with a known recurrence were recruited from two source populations: a population based cohort (U/V cohort, *n* = 458) and a randomized study (SweDCIS, *n* = 1,046).

Molecular Characteristics of primary DCIS	All DCIS with a recurrence (*n* = 266)					
	U/V cohort (*n* = 100) Type of recurrence		SweDCIS (*n* = 166) Type of recurrence		All DCIS with a recurrence (*n* = 266) Type of recurrence	
	Invasive (*n* = 55) Number	*In situ* (*n* = 45) Number	Invasive (*n* = 81) Number	*In situ* (*n* = 85) Number	Invasive (*n* = 136) Number (%)	*In situ* (*n* = 130) Number (%)
ER (*n* = 181)						
Positive	38	27	36	32	74 (81.3)	59 (65.5)
Negative	10	16	7	15	17 (18.7)	31 (34.5)
PR (*n* = 183)						
Positive	25	20	28	24	53 (55.8)	44 (50.0)
Negative	26	22	16	22	42 (44.2)	44 (50.0)
HER2 (*n* = 177)						
Positive	15	18	13	22	28 (30.4)	40 (47.1)
Negative	37	24	27	21	64 (69.6)	45 (52.9)
EGFR (*n* = 143)						
Positive	10	16	14	19	24 (32.0)	35 (51.5)
Negative	33	18	18	15	51 (68.0)	33 (48.5)
CK5/6 (*n* = 170)						
Positive	42	32	40	46	82 (94.3)	78 (94.0)
Negative	3	4	2	1	5 (5.7)	5 (6.0)
KI67 (*n* = 146)						
High	15	11	13	16	28 (37.3)	27 (38.0)
Low	32	25	15	19	47 (62.7)	44 (62.0)
Subgroups based on IHC (*n* = 266)						
ER+/HER2−	10	6	8	9	51 (37.5)	36 (28.0)
ER+/HER2+	28	19	23	17	18 (13.2)	15 (11.5)
ER−/HER2+	4	11	4	10	8 (5.9)	21 (16.2)
**ER−/HER2−/CK+ or EFGR+	3	5	3	3	6 (4.4)	8 (6.2)
Unknown	10	4	43	46	53 (39.0)	58 (44.6)

**We used the classification for basal-like DCIS published by Livasy et al., 2007 [22], and also used in an earlier paper by us [37].

**Table 3:** Univariate and multivariate analysis of the associations between baseline clinical-, histopathological-, and molecular characteristics and the risk for a recurrence being invasive carcinoma compared to *in situ *carcinoma, among women with a primary DCIS and a known recurrence in the Uppsala/Västerås cohort, in the SweDCIS randomized study and in the two groups pooled together (*n* = 266).

	Risk of a recurrence after DCIS being invasive compared to a new *in situ *					
	U/V cohort (*n* = 100)		SweDCIS (*n* = 166)		All DCIS with a recurrence (*n* = 266)	
	Univariate* OR (95% CI)	Multivariate^†^ OR (95% CI)	Univariate* OR (95% CI)	Multivariate^†^ OR (95% CI)	Univariate* OR (95% CI)	Multivariate^†^ OR (95% CI)
Mode of detection						
Screening	1.0	1.0	1.0	1.0	1.0	1.0
Clinically	1.82 (0.66–5.08)	1.83 (0.61–5.5)	1.64 (0.83–3.27)	1.71 (0.85–3.46)	1.72 (0.98–3.01)	1.80 (1.02–3.19)
Tumor size						
≤15 mm	1.0	1.0	1.0	1.0	1.0	1.0
>15 mm or multifocal	0.31 (0.12–0.84)	0.32 (0.11–0.93)	0.84 (0.41–1.63)	0.84 (0.32–1.57)	0.55 (0.33–0.93)	0.54 (0.32–0.92)
Type of surgery						
Breast conserving surgery	1.0	—	—	—	1.0	—
Mastectomy	0.88 (0.20–3.88)	—	—	—	1.13 (0.29–4.42)	—
Postoperative radiotherapy						
No	1.0	1.0	1.0	1.0	1.0	1.0
Yes	0.95 (0.39–2.34)	1.27 (0.46–3.56)	1.59 (0.80–3.20)	1.70 (0.83–3.46)	1.32 (0.76–2.27)	1.41 (0.80–2.48)
Free margins						
Yes	1.54 (0.28–8.36)	—	0.93 (0.41–2.13)		1.24 (0.69–2.22)	—
No or doubtful	1.0	—	1.0		1.0	—
Nuclear grade						
I	1.0	1.0	1.0	1.0	1.0	1.0
II	1.37 (0.35–5.4)	1.23 (0.29–5.15)	0.21 (0.02–1.87)	0.20 (0.02–1.81)	0.75 (0.26–2.12)	0.70 (0.25–2.02)
III	1.58 (0.41–6.06)	1.55 (0.38–6.37)	0.12 (0.02–1.02)	0.11 (0.01–0.96)	0.53 (0.19–1.45)	0.49 (0.18–1.35)
ER						
Negative	1.0	1.0	1.0	1.0	1.0	1.0
Positive	2.23 (0.86–5.78)	2.29 (0.87–6.03)	2.54 (0.91–7.10)	3.34 (1.10–10.2)	2.33 (1.17–4.65)	2.52 (1.24–5.10)
PR						
Negative	1.0	1.0	1.0	1.0	1.0	1.0
Positive	1.03 (0.45–2.38)	1.03 (0.44–2.39)	1.83 (0.76–4.42)	2.20 (0.83–5.40)	1.32 (0.73–2.38)	1.36 (0.75–2.47)
HER2						
Negative	1.0	1.0	1.0	1.0	1.0	1.0
Positive	0.55 (0.23–1.30)	0.60 (0.24–1.47)	0.46 (0.19–1.11)	0.42 (0.17–1.07)	0.50 (0.27–0.92)	0.48 (0.26–0.90)
EGFR						
Negative	1.0	1.0	1.0	1.0	1.0	1.0
Positive	0.35 (0.13–0.92)	0.36 (0.13–0.98)	0.62 (0.23–1.64)	0.57 (0.21–1.58)	0.45 (0.23–0.88)	0.44 (0.22–0.88)
Ki67						
Low	1.0	1.0	1.0	1.0	1.0	1.0
High	1.10 (0.41–2.97)	1.09 (0.38–3.12)	1.03 (0.38–2.82)	1.10 (0.38–3.19)	0.98 (0.50–1.94)	0.93 (0.46–1.85)
Subgroups based on IHC						
ER+/HER2−	1.0	1.0	1.0	1.0	1.0	1.0
ER+/HER2+	1.15 (0.35–3.77)	1.27 (0.37–4.39)	0.64 (0.21–2.02)	0.64 (0.20–2.02)	0.84 (0.37–1.89)	0.83 (0.37–1.88)
ER−/HER2+	0.25 (0.07–0.93)	0.26 (0.07–0.98)	0.30 (0.08–1.12)	0.22 (0.05–0.95)	0.27 (0.11–0.68)	0.24 (0.09–0.62)
**ER−/HER2−/CK5/6+ or EGFR+	0.42 (0.09–1.99)	0.41 (0.08–0.98)	0.75 (0.13–4.20)	0.75 (0.13–4.30)	0.54 (0.17–1.71)	0.52 (0.16–1.65)
Unknown	1.68 (0.46–6.17)	1.62 (0.43–6.12)	0.70 (0.33–1.48)	0.70 (0.33–1.48)	0.75 (0.42–1.34)	0.76 (0.42– 1.35)

*Adjustments for age group. ^†^Adjustments for age group, free margin, and type of surgery. **We used the classification for basal-like DCIS published by Livasy et al., 2007 [22], and also used in an earlier paper by us [37].

## Abbreviations

ER: Estrogen receptors
PR: Progesterone receptors
HER2: Human epidermal growth factor receptor 2
EGFR: Epidermal growth factor receptor
CK 5/6: Cytokeratin 5/6
TMA: Tissue microarray
IHC: Immunohistochemistry
SISH: Silver-enhanced *in situ* hybridization
OR: Odds ratio
CI: Confidence interval
NG: Nuclear grade.
